# Supplementary material for: Large-scale multi-omic biosequence transformers for modeling protein–nucleic acid interactions
Source: PLoS One. 2026 Feb 2;21(2):e0341501. doi: 10.1371/journal.pone.0341501 (PMC12863687; doi:10.1371/journal.pone.0341501)
Supplement: S1 File — (PDF) [file pone.0341501.s001.pdf]

Supporting information

S1 Appendix. OmniBioTE model architecture and training pipeline. In this section we provide a detailed description of the OmniBioTE architecture and the end-to-end training pipeline used to pretrain the model on mixed nucleic acid and protein sequences. The implementation follows a standard transformer encoder architecture with minor modifications for rotary positional encodings and maximal update parameterization (µP).

Input preprocessing and tokenization

Each training example is a single primary sequence drawn either from GenBank (nucleic acids) or UniRef100 (proteins). Input sequences are preprocessed and tokenized as follows:

1. **Sequence selection.** We sample a single raw sequence s from the mixed training corpus. For multi-omic training runs, we sample from nucleic acid and protein pools.
2. **Modality-specific tokenization.**
   - Nucleic acid sequences are tokenized with a SentencePiece tokenizer trained on GenBank entries.
   - Protein sequences are tokenized with a SentencePiece tokenizer trained on UniRef100.
   - Nucleic acid and protein vocabularies are strictly disjoint.
   - For single-character models, a disjoint single-character tokenizer was used.
3. **Context windowing.** Tokenized sequences are split or concatenated into fixed-length segments of length 1024 for BPE models or 2048 for per-residue/nucleotide models.

Encoder transformer backbone

The OmniBioTE backbone is a non-causal transformer encoder with L layers and L attention heads (each head has dimension 128), and hidden width $d = 128 \times L$. The main components are:

Token embeddings and positional encodings. A learned token embedding table $\mathbf{E} \in \mathbb{R}^{V \times d}$ maps each token index x_t to a vector $\mathbf{e}_t = \mathbf{E}[x_t]$.

Where V is the joint vocabulary size. The resulting sequence of hidden states is passed through a dropout layer and then to the transformer blocks.

RMS-style layer normalization. Each block employs an RMS normalization layer. Given an input vector $\mathbf{h} \in \mathbb{R}^d$, the layer computes

$$\mu = \frac{1}{d}\sum_{i=1}^{d} h_i, \quad \sigma = \sqrt{\frac{1}{d}\sum_{i=1}^{d}(h_i - \mu)^2 + \varepsilon},$$

and outputs

$$\text{RMSNorm}(\mathbf{h}) = \boldsymbol{\alpha} \odot \frac{\mathbf{h} - \mu}{\sigma} + \boldsymbol{\gamma},$$

where $\boldsymbol{\alpha}, \boldsymbol{\gamma} \in \mathbb{R}^d$ are learned scale and shift parameters and ε is a small constant (here 10^{-5}).

Multi-head self-attention with RoPE [1]. For each block, the self-attention layer maps an input sequence $\mathbf{X} \in \mathbb{R}^{T \times d}$ to an output $\mathbf{Y} \in \mathbb{R}^{T \times d}$:

1. A single linear projection produces concatenated queries, keys, and values:

$$[\mathbf{Q}, \mathbf{K}, \mathbf{V}] = \mathbf{X}\mathbf{W}_{\text{qkv}} \quad \mathbf{W}_{\text{qkv}} \in \mathbb{R}^{d \times 3d}.$$

2. The resulting matrices are reshaped into H attention heads of dimension $d_h = 128$:

$$\mathbf{Q}, \mathbf{K}, \mathbf{V} \in \mathbb{R}^{H \times T \times d_h}.$$

3. Rotary positional embeddings are applied to $\mathbf{Q}$ and $\mathbf{K}$. For each head and time index t, we interpret the last dimension as complex pairs and multiply by a precomputed complex sinusoid $\omega_t \in \mathbb{C}^{d_h/2}$:

$$\tilde{\mathbf{Q}}_t, \tilde{\mathbf{K}}_t = \text{RoPE}(\mathbf{Q}_t, \mathbf{K}_t; \omega_t).$$

4. Full non-causal attention is computed using PyTorch's fused scaled dot-product attention. The attention output per head is

$$\mathbf{A} = \text{Attention}(\tilde{\mathbf{Q}}, \tilde{\mathbf{K}}, \mathbf{V}),$$

where the attention logit scaling factor is set to $\alpha = 8/d$ to match µP scaling.

5. The head outputs are concatenated and projected back to the model dimension:

$$\mathbf{Y} = \text{Dropout}\left(\text{Concat}_{h=1}^{H}(\mathbf{A}^{(h)})\mathbf{W}_\text{o}\right),$$

with $\mathbf{W}_\text{o} \in \mathbb{R}^{d\times d}$.

SwiGLU MLP. Following the attention layer, each block applies a SwiGLU feed-forward network:

1. A linear layer expands the hidden dimension by a factor of 4:

$$\mathbf{U} = \mathbf{X}\mathbf{W}_\text{ff}, \quad \mathbf{W}_\text{ff} \in \mathbb{R}^{d\times 4d}.$$

2. The expanded representation is split into a value component $\mathbf{A}$ and a gating component $\mathbf{B}$ along the last dimension and passed through a SwiGLU nonlinearity.

3. A projection back to dimension d produces the MLP output, which is then passed through dropout:

$$\mathbf{Y}_\text{MLP} = \text{Dropout}(\mathbf{Z}\mathbf{W}_\text{proj}).$$

The MLP uses the same µP-compatible initialization as the attention projections: weights are initialized with standard deviation $\sigma_\text{param} = 1/\sqrt{d}$ and the output projection is additionally scaled by $1/\sqrt{2}$ because its input width is halved by SwiGLU.

Residual block structure. Each transformer block combines these components via pre-normalization and residual connections:

$$\begin{aligned}
\mathbf{h}_0 &= \mathbf{x}, \\
\mathbf{h}_1 &= \mathbf{h}_0 + \text{SelfAttention}(\text{RMSNorm}(\mathbf{h}_0)), \\
\mathbf{h}_2 &= \mathbf{h}_1 + \text{MLP}(\text{RMSNorm}(\mathbf{h}_1)).
\end{aligned}$$

After L such blocks, a final RMS normalization layer produces the encoder output $\mathbf{Z} \in \mathbb{R}^{T\times d}$.

Output head and masked language modeling objective

For pretraining, OmniBioTE is trained with a masked language modeling (MLM) objective over both modalities:

1. **Masking strategy.** For each sequence, we independently mask each token with probability $p_\text{mask} = 0.15$. Masked positions are replaced by a dedicated MASK token. The original unmasked token indices are retained as the prediction targets.

2. **Projection head.** The final hidden states $\mathbf{Z} \in \mathbb{R}^{T\times d}$ are projected to logits over the vocabulary with a single linear layer whose weights are not shared with the input embedding matrix:

$$\mathbf{L} = \mathbf{Z}\mathbf{W}_\text{lm}, \quad \mathbf{W}_\text{lm} \in \mathbb{R}^{d\times V}.$$

To maintain µP scaling, the logits are multiplied by a global factor

$$\mathbf{L}' = \left(\frac{32}{d}\right)\mathbf{L},$$

which allows learning rates to be specified in a width-invariant fashion.

3. **Loss computation.** Cross-entropy loss is computed only on masked positions. Let $\mathcal{M}$ denote the set of masked indices in the batch. The MLM loss is

$$\mathcal{L}_{\mathrm{MLM}} = -\frac{1}{|\mathcal{M}|} \sum_{(b,t)\in\mathcal{M}} \log p\left(x_{b,t} \mid x_{b,\neg t}; \theta\right),$$

where $p(\cdot)$ is the softmax over $\mathbf{L}'$ and $x_{b,\neg t}$ denotes all tokens in the sequence excluding position t.

Training

- **Batch sizing.** The global batch size B_{global} is chosen so that the token throughput approaches the memory/computation limit of the cluster. In practice we used global batch sizes in the range of 786,432–1,048,576 tokens per step.
- **Optimizer.** We use AdamW [2] and µP learning rates:
  - Embedding and unembedding layers, as well as all bias and normalization parameters, are assigned a *fixed* learning rate.
  - All remaining weights are assigned a learning rate that scales with $32/d$, and their weight decay is scaled inversely so that the effective decrease per optimization step is width-invariant.
- **Scheduler.** A OneCycleLR [3] schedule is applied over the entire training run, with an initial warmup period of 1 billion tokens and decay to the original learning rate multiplied by 10^{-5}.

This architecture and training setup is identical for all OmniBioTE sizes, with only (L, H, d) and the tokenization (BPE vs per-residue/nucleotide) varying between model families.

S2 Appendix. Predicting binding interactions between proteins and nucleic acids with AlphaFold3 and molecular dynamics. We developed and assessed a pipeline for predicting the interaction energy between proteins and nucleic acids by combining AlphaFold3 (AF3) [4] with molecular dynamics (MD) simulations performed in OpenMM version 8.1.2 [5]. Our pipeline is available at the link https://github.com/hockyg/af3_protein_nucleic_md_pipeline. We applied this pipeline to as many targets as possible from the ProNAB database studied in our binding energy experiments. Ultimately, we were able to generate structure predictions and perform MD simulations on 599 protein/nucleic acid pairs.

Given that we wanted to compare our ability to predict binding energies from sequence directly, this required us to generate bound conformations via a machine learning approach that treats both proteins and nucleic acids, and so for that we selected one of the only available options, AF3 [4]. Due to the large size of the systems and the need to rapidly evaluate interactions, we were forced to use an *implicit solvent* approach. This need was exacerbated by the fact that AF3 predicted structures often have large regions with low confidence scores that are non-compact, resulting in simulation boxes that would be intractable if filled with water (i.e. millions of atoms including solvent and ions). Using implicit solvent, the MD simulations executed for our targets ranged in size from 1195 atoms to 60832 atoms, with an average size of approximately 7653.

To approximately compute the binding energy between a protein and nucleic acid in tractable computational time, we adopted a protocol similar to the so-called MM/GBSA approach [6]. To compute the binding free energy of a complex, we need to compute

$$\Delta G = G_{\mathrm{AB}} - G_{\mathrm{A}} - G_{\mathrm{B}}, \tag{1}$$

where A and B are the separate components and the free energies on each side are averaged over a conformational ensemble. ΔG has contributions that come from the (1) direct interaction energy between the molecules, (2) the change in solvation free energy due to the difference in buried surface area, (3) and the change in configurational entropy of both parts upon binding. When using simulations with implicit solvent, effects 1 and 2 are taken into effect if we simply calculate the MD energy. The third effect due to overall changes in the conformations of the bound and unbound A and B molecules is not possible to calculate in a single simulation and requires extensive calculations beyond the scope of this work. However, conveniently, we expect that for calculations of $\Delta\Delta G$ of mutation, this term cancels out. Below, we will therefore run short

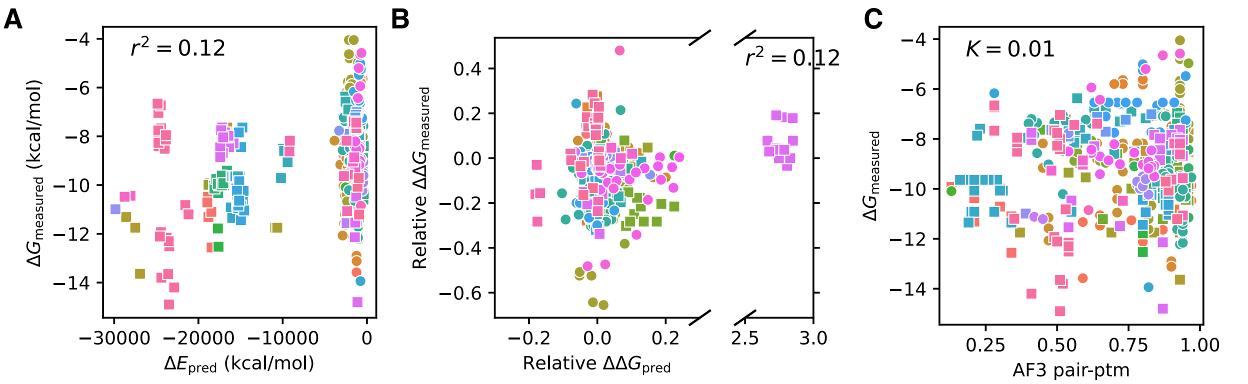

Fig 1. (**A**) Comparison of energy differences between bound and separated states using MD as described in the text with experimentally measured values. Squares denote proteins bound to DNA while circles are proteins bound to RNA. Color indicates a different protein. (**B**) Relative predicted free energy difference between binding a native and mutant nucleic acid sequence. (**C**) Comparison of experimental binding free energies with AF3 pTM scores (0-1 with 1 being a high confidence in binding prediction).

MD simulations and compute the energy of the complex as well as for separate components in order to see whether $\Delta G^{\mathrm{experiment}}$ can be predicted. *We emphasize that we do not expect this to work in general [7], and we are performing these calculations to set a baseline for our ML predictions given in the main text.*

To go from sequence to energy prediction, we start by converting entries in the ProNAB database [8] into YAML files suitable for AF3 predictions. This consists of specifying a protein chain and a nucleic acid chain (or chains in the case of a double stranded sequence). We also added 1 Mg^{2+} ion per nucleotide in case explicit divalent cations were needed for solvated MD simulations in the future. These divalent ions were removed for implicit solvent simulations performed next.

The topology of the system was built from the AF3 output CIF file using PDBFixer in OpenMM [5]. The forcefield used was Amber14 [9] with the GB2 implicit solvent model [10]. After minimizing the energy, the velocities were randomized and the system was equilibrated at $T = 300$K by running 100 ps of MD, before running 10 ns of MD using a 4 fs timestep and the `LangevinMiddle` integrator [11, 12] with hydrogen mass repartitioning to a mass of 3 amu and a drag coefficient of 1 ps^{-1}. The energies of the full complex, and the protein and nucleic acid separately were averaged over the final 5 ns of MD simulation to produce the $\Delta E_{\mathrm{pred}} = E_{AB} - E_A - E_B$ values in S1 Fig.

S1 Fig. Comparison of experimental binding energies with metrics predicted by AlphaFold3 and molecular dynamics. **A** Comparison of energy differences between bound and separated states using MD as described in the text with experimentally measured values. Squares denote proteins bound to DNA while circles are proteins bound to RNA. Color indicates a different protein. (**B**) Relative predicted free energy difference between binding a native and mutant nucleic acid sequence. (**C**) Comparison of experimental binding free energies with AF3 pTM scores (0-1 with 1 being a high confidence in binding prediction).

S1 Fig shows a scatter plot comparing ΔE_{pred} with measured binding free energies for those complexes. Multiple values are given for the same protein when different nucleic acid sequences were given in the database for mutational studies. As can be seen, both the order of magnitude is greatly different, and also there is little to no correlation as measured by the square of the Pearson correlation, r^2.

In S1 Fig B we show the results of computing a predicted $\Delta\Delta G_{\mathrm{pred}} \approx \Delta E^{\mathrm{mutant}}_{\mathrm{pred}} - \Delta E^{\mathrm{native}}_{\mathrm{pred}}$ to those from experiment. The absolute values of $\Delta\Delta G_{\mathrm{pred}}$ were much larger than for experiment again, so here we show the relative difference (i.e. scaled by the native binding free energy or energy value). Again here there is no correlation. There is also one target (p04390) which is an outlier, but there is nothing obvious suggesting why this protein shows such a large relative error compared to the others. There is also little to no correlation when removing this outlier.

We check whether the pTM confidence scores reported by AF3 [4] are correlated with the binding affinity for these complexes. This metric also has no correlation, as measured by the Spearman rank order correlation coefficient, K (S1 Fig C).

Finally, we can also consider the time required to compute these results. Simulations that we attempted ranged from 5 to 912 ns/day on a single GPU. For those simulations that completed the full 10 ns of MD, times ranged from approximately 0.01 to 0.2 hours, for a total of approximately 13.4 GPU-hours. While this already far exceeds the inference time from our ML model, this was not the time consuming part of the calculation. AF3 calculations can be split into two parts, one which involves a multiple sequence alignment (MSA), and then the actual inference [4]. While the inference step is relatively fast, the MSA step is slow and CPU bound, and was the longest part of the calculation. For these calculations, the average inference took 0.9±0.4 minutes on an A100 GPU, while the average MSA computation time took 47.5 minutes and ranged from 14 to 283 minutes on 16 CPUs, for a total of over 6700 CPU-hours.

References

1. Su J, Ahmed M, Lu Y, Pan S, Bo W, Liu Y. Roformer: Enhanced transformer with rotary position embedding. Elsevier; 2024.

2. Loshchilov I, Hutter F. Decoupled Weight Decay Regularization; 2019. Available from: https://arxiv.org/abs/1711.05101. arXiv:1711.05101.

3. Smith LN, Topin N. Super-Convergence: Very Fast Training of Neural Networks Using Large Learning Rates; 2018. Available from: https://arxiv.org/abs/1708.07120. arXiv:1708.07120.

4. Abramson J, Adler J, Dunger J, Evans R, Green T, Pritzel A, et al. Accurate structure prediction of biomolecular interactions with AlphaFold 3. Nature. 2024 jun;630(8016):493-500. Available from: https://doi.org/10.1038/s41586-024-07487-w. doi:10.1038/s41586-024-07487-w.

5. Eastman P, Galvelis R, Peláez RP, Abreu CR, Farr SE, Gallicchio E, et al. OpenMM 8: molecular dynamics simulation with machine learning potentials. J Phys Chem B. 2023;128(1):109-16.

6. Wang E, Sun H, Wang J, Wang Z, Liu H, Zhang JZ, et al. End-point binding free energy calculation with MM/PBSA and MM/GBSA: strategies and applications in drug design. Chem Rev. 2019;119(16):9478-508.

7. Roux B, Chipot C. Editorial Guidelines for Computational Studies of Ligand Binding Using MM/PBSA and MM/GBSA Approximations Wisely. J Phys Chem B. 2024;128(49):12027-9.

8. Harini K, Srivastava A, Kulandaisamy A, Gromiha MM. ProNAB: database for binding affinities of protein–nucleic acid complexes and their mutants. Nucleic Acids Research. 2021 10;50(D1):D1528-34. Available from: https://doi.org/10.1093/nar/gkab848. arXiv:https://academic.oup.com/nar/article-pdf/50/D1/D1528/42058446/gkab848.pdf. doi:10.1093/nar/gkab848.

9. Maier JA, Martinez C, Kasavajhala K, Wickstrom L, Hauser KE, Simmerling C. ff14SB: improving the accuracy of protein side chain and backbone parameters from ff99SB. J Chem Theor Comput. 2015;11(8):3696-713.

10. Nguyen H, Roe DR, Simmerling C. Improved generalized born solvent model parameters for protein simulations. J Chem Theor Comput. 2013;9(4):2020-34.

11. Leimkuhler B, Matthews C. Robust and efficient configurational molecular sampling via Langevin dynamics. J Chem Phys. 2013;138(17).

12. Zhang Z, Liu X, Yan K, Tuckerman ME, Liu J. Unified efficient thermostat scheme for the canonical ensemble with holonomic or isokinetic constraints via molecular dynamics. J Phys Chem A. 2019;123(28):6056-79.
