# Supplementary material for: Large-scale multi-omic biosequence transformers for modeling protein–nucleic acid interactions
Source: PLoS One. 2026 Feb 2;21(2):e0341501. doi: 10.1371/journal.pone.0341501 (PMC12863687; doi:10.1371/journal.pone.0341501)
Supplement: S1 Table — (DOCX) [file pone.0341501.s002.docx]

#### S1 Table.

**Training data statistics across all sequence types.**

| **Sequence Type** | (bp/residues) | (bp/residues) | (bp/residues) |
| --- | --- | --- | --- |
| DNA | 16941.82 $\pm$ 1421192.40 | 6 | 363684565 |
| mRNA | 624.75 $\pm$ 539.25 | 6 | 84308 |
| RNA | 10027.20 $\pm$ 39070.07 | 6 | 167463040 |
| cRNA | 1769.64 $\pm$ 1945.04 | 35 | 157276 |
| rRNA | 482.19 $\pm$ 266.62 | 24 | 7097 |
| ss-RNA | 3087.72 $\pm$ 4797.85 | 14 | 35911 |
| ss-DNA | 1637.48 $\pm$ 1253.90 | 17 | 34395 |
| ds-RNA | 2075.30 $\pm$ 2197.43 | 48 | 31081 |
| tRNA | 249.23 $\pm$ 349.17 | 20 | 1208 |
| ds-cRNA | 657.16 $\pm$ 970.66 | 127 | 15341 |
| ms-DNA | 15492.76 $\pm$ 19181.55 | 84 | 45513 |
| ds-mRNA | 1695.75 $\pm$ 547.50 | 1114 | 2414 |
| ms-RNA | 606.50 $\pm$ 28.50 | 578 | 635 |
| ds-rRNA | 580.00 $\pm$ 0.00 | 580 | 580 |
| peptide | 388.19 $\pm$ 379.80 | 5 | 45359 |
|  |  |  |  |
