# Supplementary material for: Large-scale multi-omic biosequence transformers for modeling protein–nucleic acid interactions
Source: PLoS One. 2026 Feb 2;21(2):e0341501. doi: 10.1371/journal.pone.0341501 (PMC12863687; doi:10.1371/journal.pone.0341501)
Supplement: S2 Table — (DOCX) [file pone.0341501.s003.docx]

#### S2 Table.

**Mean F1 scores for predicted contact maps at distance thresholds of 4 Å, 6 Å, and 8 Å.**

| Model | Mean F1 score $\pm$ standard deviation (N = 10) | | |
| --- | --- | --- | --- |
|  | 4 Å | 6 Å | 8 Å |
| **OmniBioTE** | | | |
| Small | 0.6821 $\pm$ 0.0760 | 0.7428 $\pm$ 0.0752 | 0.8134 $\pm$ 0.0772 |
| Medium | 0.6951 $\pm$ 0.0595 | 0.7585 $\pm$ 0.0584 | 0.8254 $\pm$ 0.0552 |
| Large | 0.6934 $\pm$ 0.0609 | 0.7573 $\pm$ 0.0588 | 0.8230 $\pm$ 0.0562 |
| XL | 0.6962 $\pm$ 0.0546 | 0.7574 $\pm$ 0.0520 | 0.8264 $\pm$ 0.0501 |
| **OmniBioTE (per-residue/nucleotide)** | | | |
| Small | 0.8287 $\pm$ 0.1513 | 0.8675 $\pm$ 0.1410 | 0.8987 $\pm$ 0.1148 |
| Medium | 0.8773 $\pm$ 0.0789 | 0.9077 $\pm$ 0.0780 | 0.9286 $\pm$ 0.0678 |
| Large | 0.8789 $\pm$ 0.0792 | 0.9105 $\pm$ 0.0747 | 0.9310 $\pm$ 0.0651 |
| XL | 0.8796 $\pm$ 0.0740 | 0.9116 $\pm$ 0.0722 | 0.9312 $\pm$ 0.0620 |
| **LucaOne** | | | |
| LucaOne | 0.7680 $\pm$ 0.1869 | 0.8270 $\pm$ 0.1546 | 0.8713 $\pm$ 0.1231 |
