# Supplementary material for: Large-scale multi-omic biosequence transformers for modeling protein–nucleic acid interactions
Source: PLoS One. 2026 Feb 2;21(2):e0341501. doi: 10.1371/journal.pone.0341501 (PMC12863687; doi:10.1371/journal.pone.0341501)
Supplement: S3 Table — (DOCX) [file pone.0341501.s004.docx]

#### S3 Table.

**OmniBioTE performance across all 10-folds of the Pronab mutation benchmark as measured in Pearson correlation coefficient (PCC) and mean absolute error (MAE).**

| Model | $\Delta G$ PCC | $\Delta G$ MAE |
| --- | --- | --- |
| OmniBioTE-small | 0.255 $\pm$ 0.095 | 1.68 $\pm$ 0.22 |
| OmniBioTE-medium | 0.372 $\pm$ 0.073 | 1.57 $\pm$ 0.20 |
| OmniBioTE-large | 0.346 $\pm$ 0.079 | 1.60 $\pm$ 0.21 |
| OmniBioTE-XL | **0.407** $\pm$ **0.098** | **1.56** $\pm$ **0.23** |
| OmniBioTE-small (per-nucleotide/residue) | 0.227 $\pm$ 0.122 | 1.72 $\pm$ 0.20 |
| OmniBioTE-medium (per-nucleotide/residue) | 0.335 $\pm$ 0.065 | 1.63 $\pm$ 0.22 |
| OmniBioTE-large (per-nucleotide/residue) | 0.299 $\pm$ 0.127 | 1.63 $\pm$ 0.29 |
| OmniBioTE-XL (per-nucleotide/residue) | 0.378 $\pm$ 0.088 | 1.56 $\pm$ 0.25 |
| Nuc+ProtBioTE-small | 0.071 $\pm$ 0.112 | 1.71 $\pm$ 0.24 |
| Nuc+ProtBioTE-medium | 0.081 $\pm$ 0.116 | 1.83 $\pm$ 0.32 |
| Nuc+ProtBioTE-large | 0.039 $\pm$ 0.134 | 1.83 $\pm$ 0.29 |
| Nuc+ProtBioTE-XL | 0.077 $\pm$ 0.140 | 1.90 $\pm$ 0.29 |
| LucaOne | 0.200 $\pm$ 0.16 | 2.429 $\pm$ 0.293 |
| DeePNAP | 0.100 $\pm$ 0.111 | 2.442 $\pm$ 0.821 |
| AlphaFold3 + simulation | 0.332 | – |
