# Supplementary material for: Large-scale multi-omic biosequence transformers for modeling protein–nucleic acid interactions
Source: PLoS One. 2026 Feb 2;21(2):e0341501. doi: 10.1371/journal.pone.0341501 (PMC12863687; doi:10.1371/journal.pone.0341501)
Supplement: S4 Table — (DOCX) [file pone.0341501.s005.docx]

#### S4 Table.

**GUE Results (Epigenetics): Histone Modification Benchmarks (Part 1). Values represent the Matthews correlation coefficient of the predictions.**

| Model | H3 | H3K14ac | H3K36me3 | H3K4me1 | H3K4me2 | H3K4me3 |
| --- | --- | --- | --- | --- | --- | --- |
| OmniBioTE-small | 77.01 | 58.23 | 59.42 | 51.83 | 33.62 | 37.89 |
| OmniBioTE-medium | 79.75 | 66.40 | 68.01 | 60.03 | 49.56 | 55.02 |
| OmniBioTE-large | 80.64 | 67.31 | 69.48 | 59.04 | 46.64 | 55.33 |
| OmniBioTE-XL | 82.11 | 67.34 | 70.22 | 58.14 | 52.45 | 57.43 |
|  |  |  |  |  |  |  |
| OmniBioTE-small (per-nucleotide) | 77.85 | 53.96 | 60.93 | 54.67 | 30.32 | 30.32 |
| OmniBioTE-medium (per-nucleotide) | 80.76 | 56.94 | 63.58 | 54.69 | 32.52 | 45.27 |
| OmniBioTE-large (per-nucleotide) | 82.64 | 71.52 | 69.89 | 62.42 | 57.56 | 59.33 |
| OmniBioTE-XL (per-nucleotide) | 80.47 | 59.27 | 66.20 | 54.59 | 45.71 | 47.16 |
|  |  |  |  |  |  |  |
| NucBioTE-small | 76.93 | 53.83 | 56.46 | 46.81 | 36.11 | 40.34 |
| NucBioTE-medium | 75.44 | 49.76 | 59.04 | 38.98 | 27.55 | 35.10 |
| NucBioTE-large | 76.51 | 53.51 | 55.45 | 47.05 | 32.68 | 40.71 |
| NucBioTE-XL | 80.81 | 66.91 | 66.44 | 55.26 | 47.20 | 57.04 |
|  |  |  |  |  |  |  |
| HyenaDNA (Nguyen et al. 2024) | 67.17 | 31.98 | 48.27 | 35.83 | 25.81 | 23.15 |
| NT-2500M-multi (Dalla-Torre et al. 2023) | 78.77 | 56.20 | 61.99 | 55.30 | 36.49 | 40.34 |
| DNABERT-2 (Zhou et al. 2024) | 78.27 | 52.57 | 56.88 | 50.52 | 31.13 | 36.27 |
| RandomMask (Liang et al. 2023) | 77.62 | 65.07 | 63.68 | 54.47 | 53.88 | 62.19 |
| LucaOne | 72.28 | 44.61 | 46.72 | 42.33 | 28.79 | 25.96 |
