# Supplementary material for: Large-scale multi-omic biosequence transformers for modeling protein–nucleic acid interactions
Source: PLoS One. 2026 Feb 2;21(2):e0341501. doi: 10.1371/journal.pone.0341501 (PMC12863687; doi:10.1371/journal.pone.0341501)
Supplement: S5 Table — (DOCX) [file pone.0341501.s006.docx]

#### S5 Table.

**GUE Results (Epigenetics): Histone Modification Benchmarks (Part 2). Values represent the Matthews correlation coefficient of the predictions.**

| Model | H3K79me3 | H3K9ac | H4 | H4ac |
| --- | --- | --- | --- | --- |
|  |  |  |  |  |
| OmniBioTE-small | 63.48 | 61.94 | 79.70 | 47.12 |
| OmniBioTE-medium | 72.99 | 68.79 | 82.50 | 62.57 |
| OmniBioTE-large | 72.57 | 67.99 | 82.50 | 63.62 |
| OmniBioTE-XL | 73.35 | 66.75 | 81.55 | 63.71 |
|  |  |  |  |  |
| OmniBioTE-small (per-nucleotide) | 63.14 | 57.13 | 81.94 | 46.86 |
| OmniBioTE-medium (per-nucleotide) | 67.62 | 59.17 | 82.54 | 52.22 |
| OmniBioTE-large (per-nucleotide) | 73.69 | 67.91 | 83.48 | 65.86 |
| OmniBioTE-XL (per-nucleotide) | 73.07 | 60.89 | 80.90 | 58.18 |
|  |  |  |  |  |
| NucBioTE-small | 63.17 | 54.31 | 78.69 | 52.12 |
| NucBioTE-medium | 62.50 | 51.78 | 79.86 | 38.15 |
| NucBioTE-large | 66.78 | 58.41 | 80.84 | 50.19 |
| NucBioTE-XL | 72.73 | 65.95 | 82.65 | 62.41 |
|  |  |  |  |  |
| HyenaDNA (Nguyen et al. 2024) | 54.09 | 50.84 | 73.69 | 38.44 |
| NT-2500M-multi (Dalla-Torre et al. 2023) | 64.70 | 56.01 | 81.67 | 49.13 |
| DNABERT-2 (Zhou et al. 2024) | 67.39 | 55.63 | 80.71 | 50.43 |
| RandomMask (Liang et al. 2023) | 72.67 | 65.02 | 79.44 | 64.22 |
| LucaOne | 59.69 | 50.82 | 76.24 | 36.70 |
