# Supplementary material for: Large-scale multi-omic biosequence transformers for modeling protein–nucleic acid interactions
Source: PLoS One. 2026 Feb 2;21(2):e0341501. doi: 10.1371/journal.pone.0341501 (PMC12863687; doi:10.1371/journal.pone.0341501)
Supplement: S6 Table — (DOCX) [file pone.0341501.s007.docx]

#### S6 Table.

**GUE Results: Human Transcription Factors and COVID. Values represent the Matthews correlation coefficient of the predictions, with the exception of the COVID variant prediction task which uses F1-score.**

| Model | Human Transcription Factors | | | | | Covid |
| --- | --- | --- | --- | --- | --- | --- |
|  | 0 | 1 | 2 | 3 | 4 |  |
|  |  |  |  |  |  |  |
| OmniBioTE-small | 65.67 | 70.07 | 56.43 | 46.36 | 65.81 | 67.93 |
| OmniBioTE-medium | 62.37 | 72.04 | 59.63 | 47.22 | 76.02 | 69.38 |
| OmniBioTE-large | 62.53 | 72.08 | 60.40 | 51.94 | 75.76 | 69.26 |
| OmniBioTE-XL | 64.82 | 69.95 | 63.75 | 55.44 | 75.65 | 68.77 |
|  |  |  |  |  |  |  |
| OmniBioTE-small (per-nucleotide) | 64.80 | 70.83 | 53.22 | 45.29 | 73.00 | 57.30 |
| OmniBioTE-medium (per-nucleotide) | 66.86 | 69.08 | 69.12 | 51.34 | 77.69 | 73.50 |
| OmniBioTE-large (per-nucleotide) | 65.77 | 70.46 | 67.49 | 51.62 | 77.74 | 76.55 |
| OmniBioTE-XL (per-nucleotide) | 66.50 | 67.82 | 62.95 | 53.32 | 76.02 | 74.11 |
|  |  |  |  |  |  |  |
| NucBioTE-small | 65.50 | 69.92 | 53.82 | 38.98 | 74.00 | 66.02 |
| NucBioTE-medium | 64.19 | 66.98 | 53.50 | 50.28 | 73.03 | 59.66 |
| NucBioTE-large | 63.50 | 65.24 | 56.67 | 41.90 | 69.28 | 67.01 |
| NucBioTE-XL | 64.78 | 68.50 | 59.15 | 43.18 | 76.83 | 67.82 |
|  |  |  |  |  |  |  |
| HyenaDNA (Nguyen et al. 2024) | 62.30 | 67.86 | 46.85 | 41.78 | 61.23 | 23.27 |
| NT-2500M-multi (Dalla-Torre et al. 2023) | 66.64 | 70.28 | 58.72 | 51.65 | 69.34 | 73.04 |
| DNABERT-2 (Zhou et al. 2024) | 71.99 | 76.06 | 66.52 | 58.54 | 77.43 | 71.02 |
| RandomMask (Liang et al. 2023) | 67.13 | 72.55 | 71.64 | 60.14 | 77.20 | – |
| LucaOne | 66.84 | 69.00 | 57.23 | 41.25 | 67.83 | 38.92 |
