# Supplementary material for: Large-scale multi-omic biosequence transformers for modeling protein–nucleic acid interactions
Source: PLoS One. 2026 Feb 2;21(2):e0341501. doi: 10.1371/journal.pone.0341501 (PMC12863687; doi:10.1371/journal.pone.0341501)
Supplement: S7 Table — (DOCX) [file pone.0341501.s008.docx]

#### S7 Table.

**GUE Results: Mouse Transcription Factors. Values represent the Matthews correlation coefficient of the predictions.**

| Model | Mouse Transcription Factors | | | | |
| --- | --- | --- | --- | --- | --- |
|  | 0 | 1 | 2 | 3 | 4 |
|  |  |  |  |  |  |
| OmniBioTE-small | 46.67 | 82.67 | 81.71 | 68.29 | 43.07 |
| OmniBioTE-medium | 56.42 | 84.94 | 79.88 | 70.78 | 47.96 |
| OmniBioTE-large | 57.38 | 84.60 | 76.33 | 78.01 | 49.70 |
| OmniBioTE-XL | 60.50 | 85.01 | 83.61 | 83.26 | 52.01 |
|  |  |  |  |  |  |
| OmniBioTE-small (per-nucleotide) | 37.79 | 82.00 | 75.62 | 71.90 | 39.93 |
| OmniBioTE-medium (per-nucleotide) | 64.07 | 85.47 | 85.39 | 80.82 | 52.33 |
| OmniBioTE-large (per-nucleotide) | 63.83 | 84.86 | 83.55 | 84.24 | 51.43 |
| OmniBioTE-XL (per-nucleotide) | 63.95 | 85.60 | 81.10 | 87.52 | 53.05 |
|  |  |  |  |  |  |
| NucBioTE-small | 48.92 | 82.95 | 73.22 | 70.83 | 41.58 |
| NucBioTE-medium | 52.62 | 82.63 | 77.76 | 69.22 | 40.76 |
| NucBioTE-large | 48.34 | 81.23 | 72.00 | 69.91 | 37.15 |
| NucBioTE-XL | 53.11 | 83.38 | 73.85 | 63.73 | 48.65 |
|  |  |  |  |  |  |
| HyenaDNA (Nguyen et al. 2024) | 35.62 | 80.50 | 65.34 | 54.20 | 19.17 |
| NT-2500M-multi (Dalla-Torre et al. 2023) | 63.31 | 83.76 | 71.52 | 69.44 | 47.07 |
| DNABERT-2 (Zhou et al. 2024) | 56.76 | 84.77 | 79.32 | 66.47 | 52.66 |
| RandomMask (Liang et al. 2023) | 55.61 | 82.72 | 77.61 | 74.06 | 49.81 |
| LucaOne | 52.33 | 82.57 | 73.44 | 57.11 | 45.17 |
