# Supplementary material for: Large-scale multi-omic biosequence transformers for modeling protein–nucleic acid interactions
Source: PLoS One. 2026 Feb 2;21(2):e0341501. doi: 10.1371/journal.pone.0341501 (PMC12863687; doi:10.1371/journal.pone.0341501)
Supplement: S8 Table — (DOCX) [file pone.0341501.s009.docx]

#### S8 Table.

**Promoter Detection performance across all promoters (All) and promoter subtypes (No TATA, TATA).**

| Model | All | Promoter Type | |
| --- | --- | --- | --- |
|  |  | No TATA | TATA |
|  |  |  |  |
| OmniBioTE-XL | 88.99 | 94.05 | 68.38 |
| OmniBioTE-L | 89.43 | 93.48 | 65.71 |
| OmniBioTE-M | 88.59 | 94.17 | 68.99 |
| OmniBioTE-S | 87.07 | 92.17 | 63.45 |
|  |  |  |  |
| OmniBioTE-XL (per-nucleotide) | 93.39 | 95.25 | 85.63 |
| OmniBioTE-L (per-nucleotide) | 94.80 | 83.37 | 70.88 |
| OmniBioTE-M (per-nucleotide) | 92.91 | 94.69 | 81.73 |
| OmniBioTE-S (per-nucleotide) | 92.41 | 93.48 | 86.99 |
|  |  |  |  |
| NucBioTE-XL | 89.50 | 93.78 | 68.20 |
| NucBioTE-L | 85.37 | 90.43 | 65.39 |
| NucBioTE-M | 83.99 | 91.60 | 65.87 |
| NucBioTE-S | 86.56 | 92.39 | 65.39 |
|  |  |  |  |
| HyenaDNA (Nguyen et al. 2024) | 47.38 | 52.24 | 5.34 |
| NT-2500M-multi (Dalla-Torre et al. 2023) | 91.01 | 94.00 | 79.43 |
| DNABERT-2 (Zhou et al. 2024) | 86.77 | 94.27 | 71.59 |
| RandomMask (Liang et al. 2023) | 92.74 | 93.40 | 84.03 |
| LucaOne | 84.50 | 91.86 | 62.12 |
