# Supplementary material for: Large-scale multi-omic biosequence transformers for modeling protein–nucleic acid interactions
Source: PLoS One. 2026 Feb 2;21(2):e0341501. doi: 10.1371/journal.pone.0341501 (PMC12863687; doi:10.1371/journal.pone.0341501)
Supplement: S9 Table — (DOCX) [file pone.0341501.s010.docx]

#### S9 Table.

**Core Promoter evaluation: performance across all promoters (All) and promoter subtypes (No TATA, TATA).**

| Model |  | Promoter Type | |
| --- | --- | --- | --- |
|  | All | No TATA | TATA |
|  |  |  |  |
| OmniBioTE-XL | 64.49 | 66.09 | 73.38 |
| OmniBioTE-L | 63.99 | 65.09 | 73.29 |
| OmniBioTE-M | 63.72 | 66.96 | 78.41 |
| OmniBioTE-S | 63.53 | 65.93 | 73.01 |
|  |  |  |  |
| OmniBioTE-XL (per-nucleotide) | 70.34 | 71.33 | 79.37 |
| OmniBioTE-L (per-nucleotide) | 70.88 | 71.78 | 84.96 |
| OmniBioTE-M (per-nucleotide) | 70.75 | 70.57 | 83.38 |
| OmniBioTE-S (per-nucleotide) | 71.83 | 70.88 | 82.43 |
|  |  |  |  |
| NucBioTE-XL | 63.11 | 65.33 | 62.51 |
| NucBioTE-L | 59.73 | 63.78 | 71.35 |
| NucBioTE-M | 63.41 | 64.61 | 71.11 |
| NucBioTE-S | 69.21 | 65.76 | 74.32 |
|  |  |  |  |
| HyenaDNA (Nguyen et al. 2024) | 36.95 | 35.38 | 72.87 |
| NT-2500M-multi (Dalla-Torre et al. 2023) | 70.33 | 71.58 | 72.97 |
| DNABERT-2 (Zhou et al. 2024) | 69.37 | 68.04 | 74.17 |
| RandomMask (Liang et al. 2023) | 70.89 | 70.24 | 76.65 |
| LucaOne | 60.82 | 66.93 | 75.19 |
