# Supplementary material for: Large-scale multi-omic biosequence transformers for modeling protein–nucleic acid interactions
Source: PLoS One. 2026 Feb 2;21(2):e0341501. doi: 10.1371/journal.pone.0341501 (PMC12863687; doi:10.1371/journal.pone.0341501)
Supplement: S10 Table — (DOCX) [file pone.0341501.s011.docx]

#### S10 Table.

**Secondary structure performance. In the 3-way columns, CASP12, CB513, and TS115 scores are reported; in the 8-way columns, the corresponding scores are reported. All values are measured in accuracy.**

| Model | Secondary Structure (3-way) | | | Secondary Structure (8-way) | | |
| --- | --- | --- | --- | --- | --- | --- |
|  | CASP12 | CB513 | TS115 | CASP12 | CB513 | TS115 |
|  |  |  |  |  |  |  |
| OmniBioTE-small | 0.695 | 0.733 | 0.762 | 0.568 | 0.598 | 0.640 |
| OmniBioTE-medium | 0.717 | 0.784 | 0.794 | 0.600 | 0.642 | 0.680 |
| OmniBioTE-large | 0.722 | 0.786 | 0.801 | 0.591 | 0.646 | 0.674 |
| OmniBioTE-XL | 0.708 | 0.798 | 0.805 | 0.582 | 0.656 | 0.681 |
|  |  |  |  |  |  |  |
| OmniBioTE-small (per-residue) | 0.721 | 0.757 | 0.787 | 0.585 | 0.616 | 0.669 |
| OmniBioTE-medium (per-residue) | 0.746 | 0.813 | 0.820 | 0.619 | 0.678 | 0.707 |
| OmniBioTE-large (per-residue) | 0.749 | 0.819 | 0.825 | 0.630 | 0.685 | 0.705 |
| OmniBioTE-XL (per-residue) | 0.751 | 0.822 | 0.828 | 0.615 | 0.688 | 0.716 |
|  |  |  |  |  |  |  |
| ProtBioTE-small | 0.707 | 0.769 | 0.782 | 0.568 | 0.626 | 0.667 |
| ProtBioTE-medium | 0.717 | 0.784 | 0.794 | 0.600 | 0.642 | 0.680 |
| ProtBioTE-large | 0.767 | 0.822 | 0.828 | 0.591 | 0.646 | 0.674 |
| ProtBioTE-XL | 0.764 | 0.827 | 0.831 | 0.642 | 0.691 | 0.717 |
|  |  |  |  |  |  |  |
| ESM2-t6-8M | 0.702 | 0.731 | 0.658 | 0.590 | 0.586 | 0.658 |
| ESM2-t12-35M | 0.730 | 0.773 | 0.805 | 0.607 | 0.631 | 0.690 |
| ESM2-t30-150M | 0.753 | 0.802 | 0.716 | 0.634 | 0.668 | 0.716 |
| ESM2-t33-650M | 0.780 | 0.831 | 0.843 | 0.667 | 0.700 | 0.733 |
| ESM2-t36-3B | 0.781 | 0.826 | 0.842 | 0.668 | 0.701 | 0.740 |
| LucaOne | 0.700 | 0.720 | 0.755 | 0.578 | 0.569 | 0.630 |
| TAPE-Transformer | 0.710 | 0.730 | 0.770 | 0.590 | 0.590 | 0.640 |
| TAPE-ResNet | 0.700 | 0.750 | 0.780 | 0.570 | 0.590 | 0.660 |
| TAPE-LSTM | 0.720 | 0.750 | 0.780 | 0.580 | 0.590 | 0.640 |
| Supervised (Bepler & Berger, 2019) | 0.700 | 0.730 | 0.760 | 0.570 | 0.580 | 0.650 |
| UniRep (Alley et al., 2019) | 0.720 | 0.730 | 0.770 | 0.590 | 0.570 | 0.630 |
