# Supplementary material for: Large-scale multi-omic biosequence transformers for modeling protein–nucleic acid interactions
Source: PLoS One. 2026 Feb 2;21(2):e0341501. doi: 10.1371/journal.pone.0341501 (PMC12863687; doi:10.1371/journal.pone.0341501)
Supplement: S11 Table — (DOCX) [file pone.0341501.s012.docx]

#### S11 Table.

**Remote homology (Fold, Superfamily, Family) classification performance measured in accuracy and regression performance (Fluorescence, Stability) measured in Spearman’s correlation coefficient.**

| Model | Fold | Superfamily | Family | Fluorescence | Stability |
| --- | --- | --- | --- | --- | --- |
|  |  |  |  |  |  |
| OmniBioTE-small | 0.208 | 0.906 | 0.362 | 0.666 | 0.686 |
| OmniBioTE-medium | 0.219 | 0.965 | 0.454 | 0.655 | 0.722 |
| OmniBioTE-large | 0.226 | 0.971 | 0.455 | 0.660 | 0.671 |
| OmniBioTE-XL | 0.242 | 0.970 | 0.482 | 0.659 | 0.689 |
|  |  |  |  |  |  |
| OmniBioTE-small (per-residue) | 0.201 | 0.914 | 0.342 | 0.659 | 0.700 |
| OmniBioTE-medium (per-residue) | 0.231 | 0.966 | 0.475 | 0.587 | 0.689 |
| OmniBioTE-large (per-residue) | 0.240 | 0.972 | 0.512 | 0.662 | 0.711 |
| OmniBioTE-XL (per-residue) | 0.223 | 0.973 | 0.470 | 0.539 | 0.699 |
|  |  |  |  |  |  |
| ProtBioTE-small | 0.194 | 0.951 | 0.406 | 0.666 | 0.702 |
| ProtBioTE-medium | 0.219 | 0.965 | 0.454 | 0.655 | 0.722 |
| ProtBioTE-large | 0.226 | 0.971 | 0.455 | 0.666 | 0.683 |
| ProtBioTE-XL | 0.241 | 0.972 | 0.463 | 0.663 | 0.654 |
|  |  |  |  |  |  |
| ESM2-t6-8M | 0.240 | 0.911 | 0.439 | 0.663 | 0.660 |
| ESM2-t12-35M | 0.288 | 0.961 | 0.574 | 0.673 | 0.723 |
| ESM2-t30-150M | 0.272 | 0.978 | 0.601 | 0.672 | 0.761 |
| ESM2-t33-650M | 0.231 | 0.965 | 0.530 | 0.665 | 0.720 |
| ESM2-t36-3B | 0.249 | 0.970 | 0.542 | 0.654 | 0.774 |
| LucaOne | 0.266 | 0.949 | 0.487 | 0.639 | 0.703 |
| TAPE-Transformer | 0.21 | 0.88 | 0.34 | 0.68 | 0.73 |
| TAPE-ResNet | 0.26 | 0.92 | 0.43 | 0.67 | 0.69 |
| TAPE-LSTM | 0.17 | 0.77 | 0.31 | 0.21 | 0.73 |
| Supervised (Bepler & Berger, 2019) | 0.17 | 0.79 | 0.20 | 0.33 | 0.64 |
| UniRep (Alley et al., 2019) | 0.23 | 0.87 | 0.38 | 0.67 | 0.73 |
