# Supplementary material for: Large-scale multi-omic biosequence transformers for modeling protein–nucleic acid interactions
Source: PLoS One. 2026 Feb 2;21(2):e0341501. doi: 10.1371/journal.pone.0341501 (PMC12863687; doi:10.1371/journal.pone.0341501)
Supplement: S12 Table — (DOCX) [file pone.0341501.s013.docx]

#### S12 Table.

**Contact evaluation performance, reporting Contacts P@L for long- and medium-range contacts. All values are the computed precision of the predictions.**

| Model | Contacts P@L (long) | Contacts P@L (medium) |
| --- | --- | --- |
|  |  |  |
| OmniBioTE-small | 0.286 | 0.339 |
| OmniBioTE-medium | 0.237 | 0.350 |
| OmniBioTE-large | 0.280 | 0.371 |
| OmniBioTE-XL | 0.300 | 0.334 |
|  |  |  |
| OmniBioTE-small (per-residue) | 0.544 | 0.682 |
| OmniBioTE-medium (per-residue) | 0.467 | 0.614 |
| OmniBioTE-large (per-residue) | 0.636 | 0.725 |
| OmniBioTE-XL (per-residue) | 0.755 | 0.789 |
|  |  |  |
| ProtBioTE-small | 0.307 | 0.373 |
| ProtBioTE-medium | 0.386 | 0.406 |
| ProtBioTE-large | 0.302 | 0.347 |
| ProtBioTE-XL | 0.318 | 0.394 |
|  |  |  |
| ESM2-t6-8M | 0.521 | 0.609 |
| ESM2-t12-35M | 0.506 | 0.676 |
| ESM2-t30-150M | 0.515 | 0.654 |
| ESM2-t33-650M | 0.765 | 0.822 |
| ESM2-t36-3B | 0.753 | 0.819 |
| LucaOne | 0.365 | 0.556 |
| TAPE-Transformer | 0.17 | 0.19 |
| TAPE-ResNet | 0.20 | 0.20 |
| TAPE-LSTM | 0.10 | 0.18 |
| Supervised [11] | 0.18 | 0.22 |
| UniRep [12] | 0.17 | 0.17 |
