# Supplementary material for: Large-scale multi-omic biosequence transformers for modeling protein–nucleic acid interactions
Source: PLoS One. 2026 Feb 2;21(2):e0341501. doi: 10.1371/journal.pone.0341501 (PMC12863687; doi:10.1371/journal.pone.0341501)
Supplement: S13 Table — (DOCX) [file pone.0341501.s014.docx]

#### S13 Table.

**Performance on the structural prediction tasks in the ProteinGLUE dataset. Values represent the accuracy of the predictions.**

| Model | SS3 | SS8 | SS3 CB513 | SS8 CB513 |
| --- | --- | --- | --- | --- |
|  |  |  |  |  |
| OmniBioTE-small | 77.1 | 64.9 | 77.6 | 63.0 |
| OmniBioTE-medium | 81.3 | 69.0 | 82.9 | 68.5 |
| OmniBioTE-large | 82.0 | 69.8 | 83.4 | 69.7 |
| OmniBioTE-XL | 82.7 | 70.7 | 87.0 | 72.0 |
|  |  |  |  |  |
| OmniBioTE-small (per-residue) | 76.7 | 64.5 | 76.4 | 62.6 |
| OmniBioTE-medium (per-residue) | 81.8 | 69.4 | 82.9 | 69.9 |
| OmniBioTE-large (per-residue) | 82.5 | 70.6 | 83.0 | 69.5 |
| OmniBioTE-XL (per-residue) | 82.8 | 71.1 | 83.5 | 73.0 |
|  |  |  |  |  |
| ProtBioTE-small | 79.8 | 67.3 | 81.3 | 67.0 |
| ProtBioTE-medium | 84.1 | 72.3 | 87.8 | 72.8 |
| ProtBioTE-large | 84.9 | 73.0 | 86.5 | 73.8 |
| ProtBioTE-XL | 85.4 | 74.3 | 88.8 | 75.0 |
| ESM2-t6-8M | 76.0 | 63.8 | 73.4 | 58.7 |
| ESM2-t12-35M | 79.9 | 67.8 | 77.3 | 63.0 |
| ESM2-t30-150M | 83.0 | 71.7 | 81.0 | 67.6 |
| ESM2-t33-650M | 85.3 | 83.0 | 83.0 | 70.4 |
| ESM2-t36-3B | 85.6 | 75.1 | 82.8 | 70.5 |
| LucaOne | 75.5 | 62.8 | 73.2 | 58.2 |
