# Supplementary material for: Large-scale multi-omic biosequence transformers for modeling protein–nucleic acid interactions
Source: PLoS One. 2026 Feb 2;21(2):e0341501. doi: 10.1371/journal.pone.0341501 (PMC12863687; doi:10.1371/journal.pone.0341501)
Supplement: S14 Table — (DOCX) [file pone.0341501.s015.docx]

#### S14 Table.

**Performance on the remaining tasks in the ProteinGLUE dataset.**

| **Model** | **Protein-protein interaction (AUCROC)** | **Hydrophobic patch rank (PCC)** | **Epitope detection (AUCROC)** | **Solvent accessibility (PCC)** | **Buried-residue prediction (Accuracy)** |
| --- | --- | --- | --- | --- | --- |
| **OmniBioTE** | | | | | |
| OmniBioTE-small | 0.580 | 0.264 | 0.502 | 0.615 | 86.6 |
| OmniBioTE-medium | 0.618 | 0.239 | 0.616 | 0.657 | 88.0 |
| OmniBioTE-large | 0.591 | 0.237 | 0.559 | 0.642 | 87.9 |
| OmniBioTE-XL | 0.608 | 0.265 | 0.595 | 0.565 | 88.1 |
| **OmniBioTE (per-residue)** | | | | | |
| OmniBioTE-small (per-residue) | 0.532 | 0.145 | 0.628 | 0.598 | 80.0 |
| OmniBioTE-medium (per-residue) | 0.568 | 0.114 | 0.573 | 0.623 | 82.8 |
| OmniBioTE-large (per-residue) | 0.641 | 0.133 | 0.554 | 0.659 | 83.3 |
| OmniBioTE-XL (per-residue) | 0.610 | 0.075 | 0.590 | 0.617 | 83.3 |
| **ProtBioTE** | | | | | |
| ProtBioTE-small | 0.585 | 0.272 | 0.536 | 0.624 | 87.2 |
| ProtBioTE-medium | 0.619 | 0.304 | 0.515 | 0.654 | 88.6 |
| ProtBioTE-large | 0.599 | 0.074 | 0.610 | 0.554 | 88.8 |
| ProtBioTE-XL | 0.617 | 0.284 | 0.562 | 0.593 | 89.2 |
| **Baselines** | | | | | |
| ESM2-t6-8M | 0.617 | 0.303 | 0.517 | 0.676 | 79.7 |
| ESM2-t12-35M | 0.536 | 0.211 | 0.503 | 0.711 | 82.0 |
| ESM2-t30-150M | 0.659 | 0.200 | 0.504 | 0.749 | 83.6 |
| ESM2-t33-650M | 0.653 | 0.202 | 0.523 | 0.769 | 84.8 |
| ESM2-t36-3B | 0.653 | 0.177 | 0.514 | 0.752 | 85.3 |
| LucaOne | 0.597 | 0.275 | 0.502 | 0.663 | 79.4 |
